# Review of Central-Eastern European Propagation and Larvae Nursing Method for Common Carp (*Cyprinus carpio* L.)

**DOI:** 10.3390/life13122334

**Published:** 2023-12-12

**Authors:** Laszlo Horvath, Arpad Hegyi, Kinga Katalin Lefler, Balazs Csorbai, Eva Kovacs, Tamas Szabo, Tamas Muller, Bela Urbanyi

**Affiliations:** 1 Institute of Aquaculture and Environmental Safety, Hungarian University of Agriculture and Life Sciences (MATE), 2100 Godollo, Hungary; hegyi.arpad@uni-mate.hu (A.H.); lefler.kinga@uni-mate.hu (K.K.L.); csorbai.balazs@uni-mate.hu (B.C.); szabo.tamas@uni-mate.hu (T.S.); muller.tamas@uni-mate.hu (T.M.); 2 Eurofish International Organisation, 1553 Copenhagen, Denmark; eva.kovacs@eurofish.dk

**Keywords:** common carp, propagation, reproduction, induced breeding, hatchery incubation, Zug jars, larvae of carp, starter feeds, zooplankton, plankton selection, juvenile nursing

## Abstract

Common carp (*Cyprinus carpio* L.) as a cultivated fish species has huge importance all over the world. According to FAO statistics, carp is the third most widely bred freshwater pond fish species; only two other Cyprinids (silver carp and grass carp) are bred in higher amounts. Carp is native all over Asia and in a large part of Europe. As a result of human intervention, at present, carp are widespread all over the world, except for the Arctic region. Carp breeding was launched in the antique period, in the ancient Chinese Empire and the Roman Empire. The presently applied method of breeding of common carp has a long evolution. From the effectiveness point of view, the propagation and early-life nursing are crucial parts of carp production, as they provide seed stocks for the further growing section. Without effective propagation, there is no intensive carp production. Nowadays, more advanced propagation methods are available all over the world; however, in the current review, only the main milestones and production efficiency of the propagation and nursing method used in the ponds of Eastern Central Europe are discussed. In the historical overview of carp reproduction, first the natural reproduction, then the semi-extensive and intensive hatchery propagation are presented and investigated in detail. The analysis focuses on the advantages and disadvantages of the method. In particular, the different important milestones of the advanced hatchery method are shown and explained. The effectiveness is proven even with practical calculations. Not only the reproduction, but the pond nursing method is also presented and discussed, concentrating on the management of evolutionarily adapted natural feeds (Zooplankton) and their effect on the survival of fish larvae.

## 1. Introduction—Historical Development of the Presented Reproduction Protocol of Fish

### 1.1. General Outlook

The human population on Earth exceeds 8 billion people; therefore, the continuous supply of food of plant and animal origin has become a most pressing task. In recent decades, the demand for nutritionally more valuable animal products has been increasing, especially in developing countries with huge populations, which poses a great challenge for the animal husbandry sectors [1]. It is particularly difficult to reconcile the high level of harmful environmental effects of animal products, including a significant emission of greenhouse gases, with the increasing social demand. Aquaculture is among the rare sectors that is able to reliably produce biologically valuable animal products while keeping harmful environmental effects at a low level [2]. This adds to the continuous expansion of aquaculture production.

Freshwater fish species, like common carp, play a leading role in the piscicultures of landlocked countries in Central Europe. Pisciculture, or the culturing of fish, is an important part of aquaculture [3]. Rearing common carp to market size in the temperate zone usually lasts for 2–3 years. The success of profitable breeding largely depends on high-quality seed stocks; this is often a limiting factor. Propagation and larvae nursing of common carp are important steps of the breeding procedure. This explains the strong professional interest focusing on the development of efficient propagation methods.

### 1.2. The Main Aim of this Review

An effective seed stock production method for common carp (*Cyprinus carpio* L.), as one of the most important farmed fish in Asia and Central Europe, is introduced, summarized and evaluated here.

One of the main conditions for common carp production that meets consumer needs is the efficient production of seed material. Seed production includes hatchery propagation as well as advanced fry rearing in ponds. This manuscript is a detailed presentation of these two steps of seed production, which are applied all over the world, including Europe and Southeast Asia. The goal of the manuscript was to present the cornerstones of these methods with the help of diagrams and photographs so that professionals from other regions could also apply and reproduce them.

### 1.3. Historical Outlook: Technical and Academic Developments

Prior to the analysis of the key points of hatchery propagation, historical development and the most important milestones of carp breeding are presented chronologically.

#### 1.3.1. Fish in Human Culture—Early Ages

**Figure 1:**
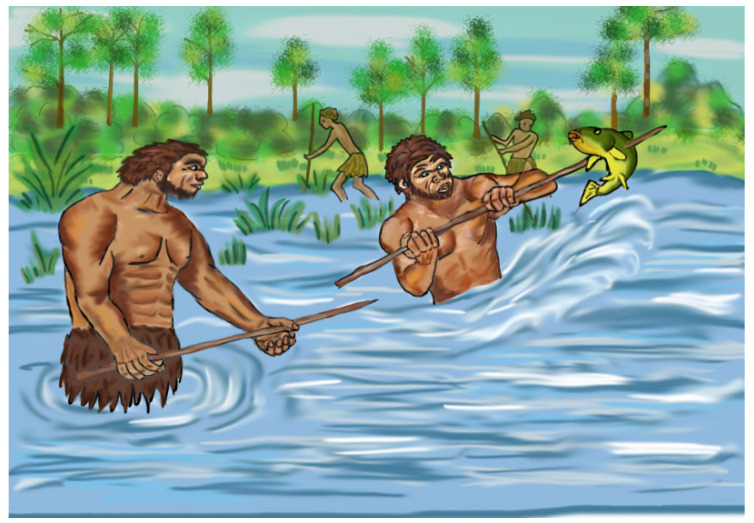
Prehistoric human population hunted for fish in shallow waters (K. Lefler).

In ancient ages, systematic breeding and propagation were not yet practiced. In prehistoric times, fish-origin foods including carp were only consumed on a temporary basis. Archaic men mainly hunted for fish when they migrated to shallow waters for spawning, or after flooding, when fish were trapped in drying waterholes (Figure 1).

At present, common carp is one of the most widely farmed freshwater fish species in Asia and Europe. The species is the oldest one domesticated all over the world from those bred for human consumption [4].

Based on early written records, it seems that the beginning of carp breeding can be connected to two great ancient cultural centers: the Chinese Empire in Asia and the Roman Empire in Europe [5]. In antiquity, our ancestors already kept carps in artificial ponds in the Chinese Empire and in stone basins called piscina in the Roman Empire. In both places, self-sustaining carp stocks were bred and developed.

#### 1.3.2. Data from the Chinese Empire

**Figure 2:**
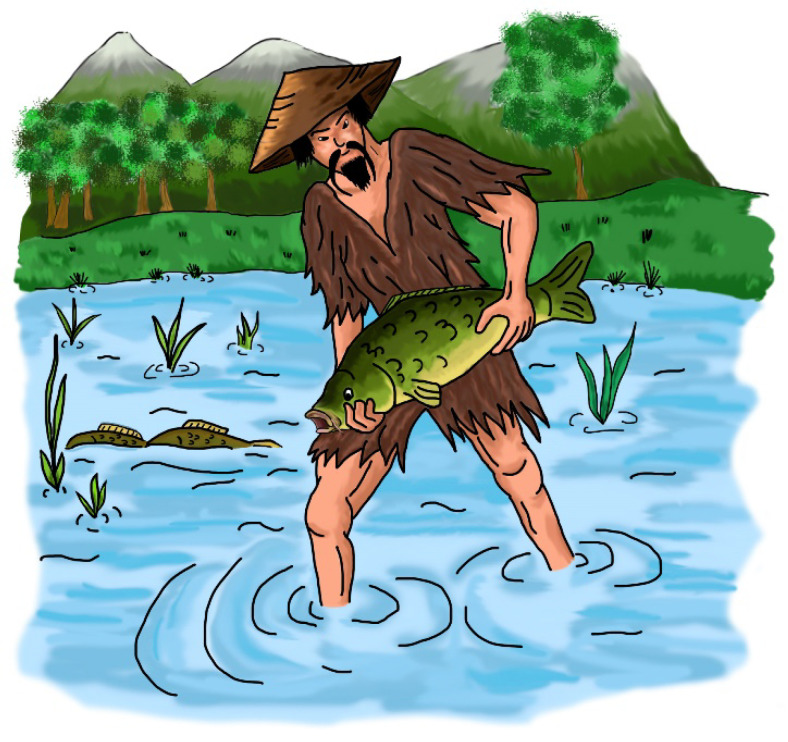
Chinese fisherman working with carp in the age of the Chinese Empire (K. Lefler).

The first carp breeding method was described by Fan Li in 463 AC, during the age of the Han Dynasty of the Chinese Empire (Figure 2).

For a long period of time, common carp stocks were maintained by natural reproduction, until the hormonal induction method was developed in the late 1950s and early 1960s [6].

At the time of the Tang Dynasty in China (600 AC), a new period was started for aquaculture. Farmers turned from monoculture systems of common carp to polycultures, made up of at least five species belonging to the family of Cyprinids. This group not only included the lacustrine common carp, but also the riverine Chinese (major) carps: grass carp (*Ctenopharyngodon Idella*), bighead carp (*Hypophthalmichthys nobilis*), silver carp (*Hypophthalmichthys molitrix*), and black carp (*Mylopharyngodon piceus*). Since then, Chinese carps have played an increasingly important role in the production structures. At the beginning, a specific method for these riverine fish species was elaborated to provide seed stock for fishponds. As these species are not able to reproduce in a lake environment, juvenile stocks were collected from natural spawning grounds and carefully transported to fish production ponds [7,8].

#### 1.3.3. Breeding Major Chinese Carps by Hormonal Induction

After induced fish reproduction was established (1950–1960s) in Far East Asian countries led by China, herbivorous Chinese carps and common carp were reproduced in pond farms by applying chorionic gonadotropin hormone, pituitary extractions, and gonadotropin-releasing hormone combined with dopamine receptor antagonists. Special small ponds or circular concrete basins with flow-through water were used for spawning, as riverine Chinese carps have non-sticky, floating (semi-pelagic) eggs, which drift freely in the water body. Thus, after hormonal treatment, Chinese carps were transferred into such small ponds, whereafter spawning floating eggs were collected in egg traps and placed into circular concrete pools for incubation and larvae rearing [7,8].

Mature broodfish of hormonally treated common carp were placed in circular or oval concrete pools with water supply, where fish could spawn following the breeding program. The surface of the freshly ovulated common carp eggs, when they were released into the water, became sticky, and they immediately adhered to the wall of the basins or to large surfaces of artificial spawning substrates placed into the pools. Carp eggs could be incubated in vertical incubators as well. Egg and larval development took place either in the spawning pool or in a different circular basin [9].

#### 1.3.4. History of Carp Breeding in Europe

In Europe, which was the other cultural center of carp production, breeding was developed in a somewhat different way. After the fall of the ancient Roman Empire, where fish were kept in piscinas, very little information was available about regular fish breeding for centuries, up until the Middle Ages. The dominant Christian religion in Europe played an important role in the development of common carp breeding. According to strict regulations, meat consumption was prohibited during long fasting periods, but this did not apply to the consumption of fish.

**Figure 3:**
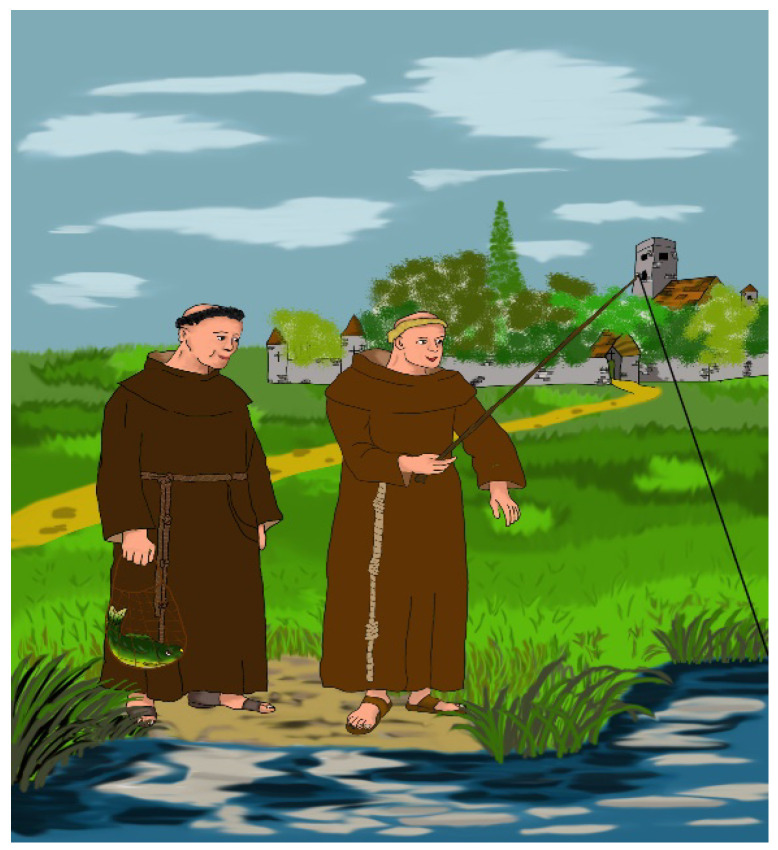
Angling monks from a monastery (K. Lefler).

As a result, fishponds were established around religious centers and Christian monasteries to ensure a continuous supply of delicious fish meals (Figure 3). In Central Europe, the old, traditional way of carp cultivation in artificially constructed fishponds first appeared in the Czech and German regions, not only in monasteries, but also as established by rich noblemen for commercial fish production. Experiences in fish husbandry were collected in a book by a Czech bishop, Janus Dubravius, around the 1560s. At that time, when information about fish propagation was very limited, fingerlings of common carp were produced by natural spawning. Then, different age groups of fish were raised in separate ponds: younger generations in smaller ponds, with older stocks in larger ones [9].

An important improvement of this period was the establishment of sluices (or monk sluices) enabling the regulation of the water level of artificial ponds. At that time, locks were prepared from wood, and in the region, these are still called “monk locks”.

By the middle of the 19th century, fishponds were already very popular, and stocks from the natural reproduction of carp were not sufficient to supply the seed demands of fishponds. To satisfy the growing interest, the first efficient and safe method of propagation was established: the so called “small-pond” spawning method. Tomas Dubits, a fisherman from Silesia, observed the natural spawning of common carp: when rivers flooded beyond their riverbanks during the spring, mature carp emerged from the main riverbed and began to reproduce on the floodplains.

#### 1.3.5. Natural Spawning Process of Carp

To understand the idea behind the Dubits method, the natural spawning of carp is briefly described first.

**Figure 4:**
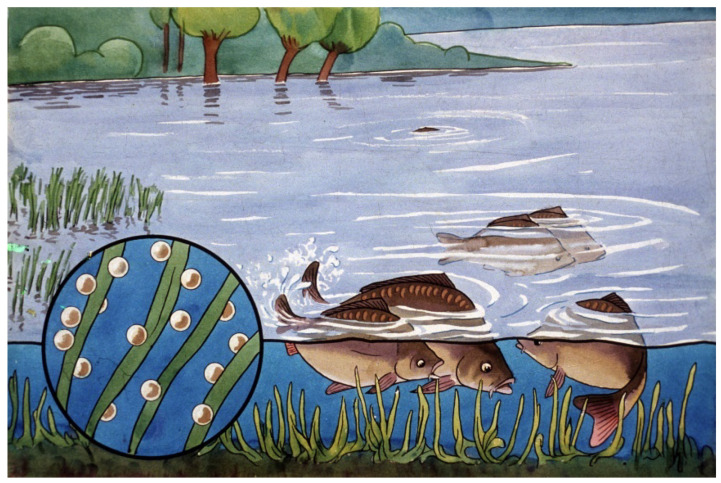
Spawning of common carp in a flooded area. Eggs stick to grass filaments [11].

During its evolution, carp, which prefers lake and river habitats, adapted to reproduce in shallow, stagnant water bodies. As a result, the surfaces of carp eggs contain a protein-like compound as a remnant of the ruptured follicular wall, which becomes sticky after the eggs are released into the water (Figure 4). The eggs are then able to attach to substrates such as grass filaments or stones (phyto-lithophilic eggs). This feature protects the eggs and later the developing embryo from sinking into the mud and dying due to a lack of oxygen [10].

**Figure 5:**
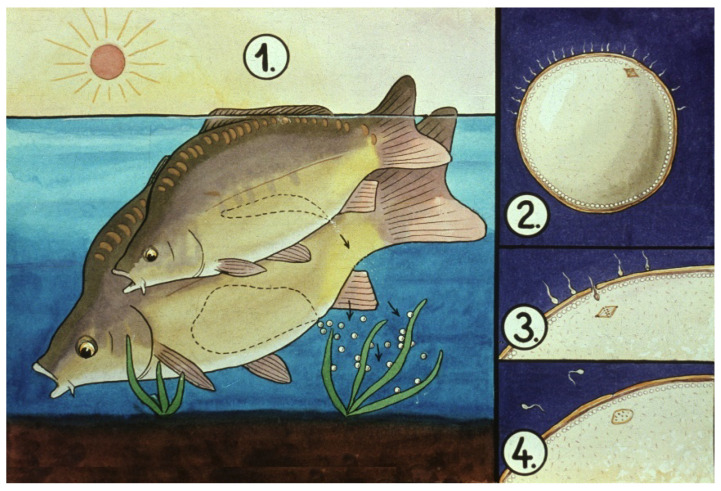
The process of carp egg fertilization in nature and in Dubits ponds. 1. The male and the female simultaneously release their gametes into the water. 2. Sperms actively find the micropyle on the egg surface. 3. One of the sperms penetrates into the opening of the micropyle, unites with the pronucleus of the oocyte, and forms a diploid zygote, which begins to divide. 4. After the first sperm moves through the micropyle, the opening closes and other sperms will not be able to fertilize the egg [12].

During natural spawning, sperm penetrate through the eggshell (through the micropyle) and fertilize the egg (Figure 5).

**Figure 6:**
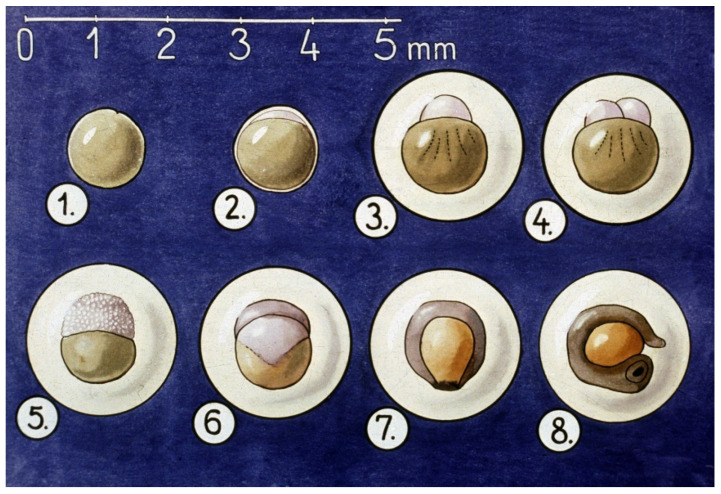
Embryogenesis of carp eggs. 1. Freshly ovulated egg. 2. The swelling process starts when the egg is released into the water. 3. After about an hour, the process of swelling is finished. A large perivitelline space forms around the egg. 4. The animal pole of the zygote starts to divide. 5. Within a few hours, the sensitive morula stage is developed. 6. Blastula stage. 7. Gastrula stage. 8. Early neurula stage. The embryo starts to move within the egg [11].

Embryogenesis of carp eggs is a three-day-long procedure (Figure 6).

**Figure 7:**
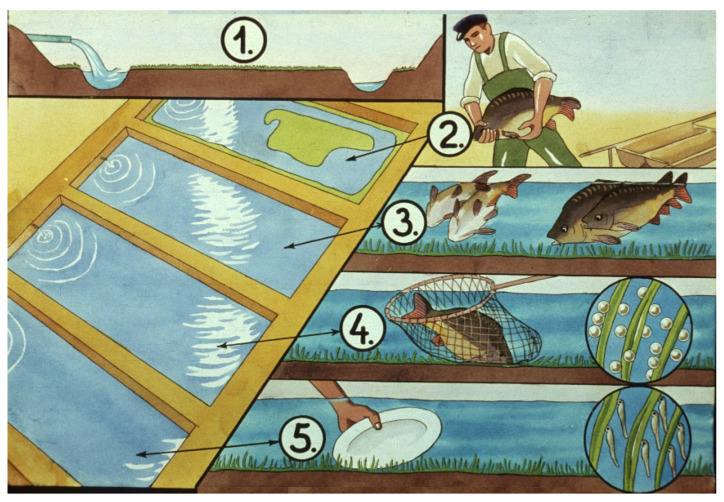
Propagation in small ponds (Dubits method). 1. Freshly filled spawning pond. 2. Broodstock is introduced. 3. Spawning takes places within days. 4. Netting broodfish. 5. Monitoring spawning results (eggs and larvae) [11].

When following the Dubits method, fish breeders simulate the environmental conditions of nature for spawning. Small, shallow spawning pools covered with grass are constructed (Figure 7). Mature carp are placed into them, where they reproduce and place their eggs on the grass fibers. In an oxygen-rich, clean water body, embryogenesis is also able to take place successfully. After hatching, carp larvae hang onto the grass.

Dubits kept the young carp fries in the small fishponds for a few days, up to the start of feeding, then collected them with a hand net and transferred them to larger, zooplankton-rich ponds for further rearing, where the size of the zooplankton was suitable to feed carp larvae. With this method, it was possible to safely produce offspring every year, independently of changing weather conditions during spring. His method was published by Otto Herman, a Hungarian scientist in an excellent book, first published in 1888 [13].

A major disadvantage of this method was that it required extensive manual labor (preparation and careful transportation of mature fish, collection, and transportation of feeding fry (so called “mosquito fish”), grassing small ponds, keeping them dry after the spawning period, etc.).

#### 1.3.6. Need for an Advanced Method

At the beginning of the 19th century, fish-farm managers realized that even with the safe Dubits method, they were not able to satisfy the growing demand for fish fingerlings, and the lack of seed stocks became permanent. The development of an even more efficient reproduction method was required; thus, intensive research started in the 1950s.

#### 1.3.7. Improving Technical Background

In continental Europe, not only warm-water carp breeding, but also cold-water fish cultures started to develop in the middle of the 18th century. Certain species of cold-water fish (including trout, a freshwater Salmonidae, which can mainly be reproduced in mountainous regions) became popular.

Propagation and breeding of trout (*Salmo trutta*), Northern pike (*Esox lucius*) and Coregonid fish started. Stefan Ludwig Jacobi (1711–1784) was a German fish breeder working in Westphalia. He was the first person who elaborated a method for the artificial propagation of trout in 1763–1765. Jacobi simulated the natural conditions of spawning: he released trout eggs into fresh water and then pressed the sperm onto it (“wet fertilization”). By this method, the fertilization rate was low, only 20% [14].

Years later, in 1843, Remy and Gehin in France, independently from Jacobi, rediscovered the artificial reproduction of trout. The French government recognized the importance of this method and built a large fish hatchery in the town of Huningue, France in 1852. This was the first large-scale industrial indoor “factory” producing fish fry all over the world [15,16]. A year later, the method was introduced into the United States by Garlick [17].

In another part of the world, in Russia, Vransky started a new way of egg fertilization: he collected separately dry salmon eggs and sperm, and after mixing them, added water to the mixture (1856) [18]. The method is called “dry fertilization”. By using this technique, fertility increased up to even 90% [14]. Nowadays, the method of dry fertilization is widely used for the artificial reproduction of different fish species all over the world.

Following artificial fertilization, the next step was the incubation of fertilized eggs in an environment close to optimal, with a high level of dissolved oxygen. For non-sticky trout egg incubation, many different types of incubators can be used—e.g., different box types or some vertical types of hatching jars. Vertical incubation of fish eggs is very economical because a large number of eggs are kept in a small place. This type of incubation is enhanced everywhere in fish hatcheries. One of the most popular incubators the Zug jar or Weiss jar elaborated in Zug Canton (Switzerland). The original hatching glass, which was built in 1882, is exhibited in a fishing museum in Zug. It was used to release the progeny of cold-water fish into the surrounding lakes, including Lake Zug [19].

Besides Zug jars, another popular vertical incubator is the McDonald-type hatching jar from the United States [20] in which different non-stick fish eggs can be incubated. Flowing water moves fish eggs very gently in these jars, so they are also suitable for hatching sensitive fish eggs with excellent results.

**Figure 8:**
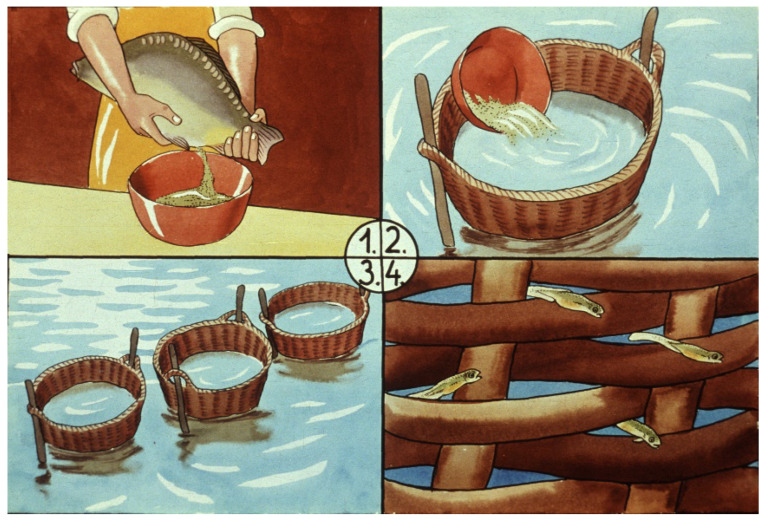
Carp egg incubation in a basket. 1. Stripping of broodfish, followed immediately by fertilization. 2. Releasing fertilized eggs into a basket. 3. Fixed baskets in open water. 4. Young carp fries swim out of the basket [11].

Due to the stickiness of common carp eggs, their incubation was not possible in such vertical flow-through systems. Therefore, after fertilization, these eggs were spread in baskets where they were stuck on the wall of the basket or other available fine surfaces [21]. After hatching, young fish could swim out from the safe incubation place. A disadvantage of this technique was the high risk of strong fungal infection: the aquatic fungus Saprolegnia could infect the eggs and kill most of them in natural water ecosystems (Figure 8).

Woynarovich from Hungary [21] and Probst from Germany [22] published a semi-artificial carp propagation method in the early 1950s, spreading sticky eggs on artificial nests (e.g., baskets: Figure 8).

To summarize: fish hatcheries working with vertical incubators (Zug jars) were not suitable for the incubation of sticky roe like carp eggs. Difficulties arise from properties of substratophile eggs. If carp eggs are released into the water immediately after fertilization, the eggs form large clumps, with all of them sticking together.

**Figure 9:**
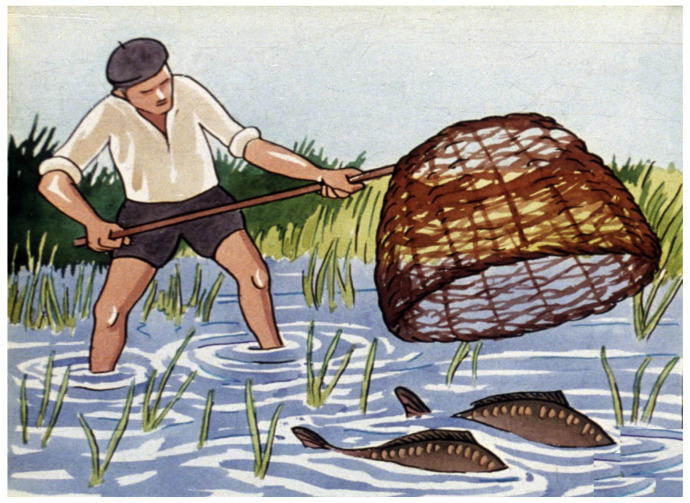
Catching adult carps during spawning to collect freshly ovulated eggs [11].

When common carp was propagated in a hatchery, another problem appeared: It was very difficult to obtain fresh carp eggs. Only mature carps could be caught during the spawning season, and natural reproduction lasts only for a few days within a year, so the amounts of freshly collected carp eggs were very limited (Figure 9).

Finally, researchers managed to resolve the two problems: eliminating the stickiness of eggs and inducing a programmed ovulation of mature common carp females by using hormonal induction.

#### 1.3.8. Elimination of the Adhesive Characteristic of Carp Eggs

Intensive practical work started around the 1960s to eliminate adhesive compounds from common carp eggs [23]. Researchers managed to successfully use fine clay suspension, diluted cow milk, and protease enzyme treatment, but finally, a simple table salt-urea solution proved to be the most suitable method and became very popular, developed, and published by Woynarovich (1962) [24]. Currently, this method is widely used in most carp hatcheries of Europe. A detailed description of the process is provided later.

#### 1.3.9. Hormonal Induction of Fish Ovulation

For understanding the complicated physiological characteristic of a mature fish, clarifying the reproductive difference between poikilotherm fish and homeotherm mammals must be discussed.

**Figure 10:**
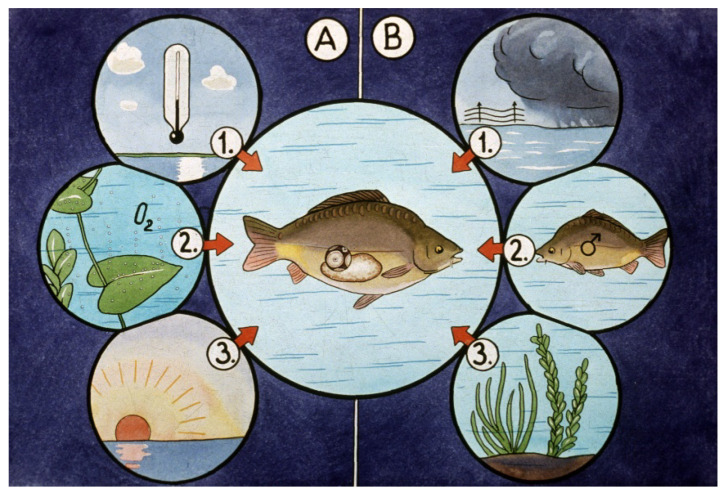
Environmental factors influencing carp reproduction. (**A**) Basic factors affecting physiological processes, including reproductivity. 1. Temperature, 2. Dissolved oxygen, 3. Light. (**B**) Inducing factors launching ovulation and spermiation of mature carp. 1. Meteorological fronts, 2. Presence of both sexes of fish 3 Surfaces where adhesive eggs can be laid [11].

Thanks to the stable internal temperature (homeostasis) of homeotherm animals, the cyclic reproductive activity is independent from the temperature of the surrounding environment, while in poikilotherm animals like fish, the temperature of the environment, together with other external factors, strongly influences reproduction. Oocyte maturation occurs at temperatures the fish was adapted to during evolution. While common carp needs around 20 °C for a normal oogenesis, trout only needs less than 10 °C. When gametogenesis (both spermatogenesis and oogenesis) is completed, the reproductive process stops (dormant stage), waiting for favorable outside conditions to appear (Figure 10).

**Figure 11:**
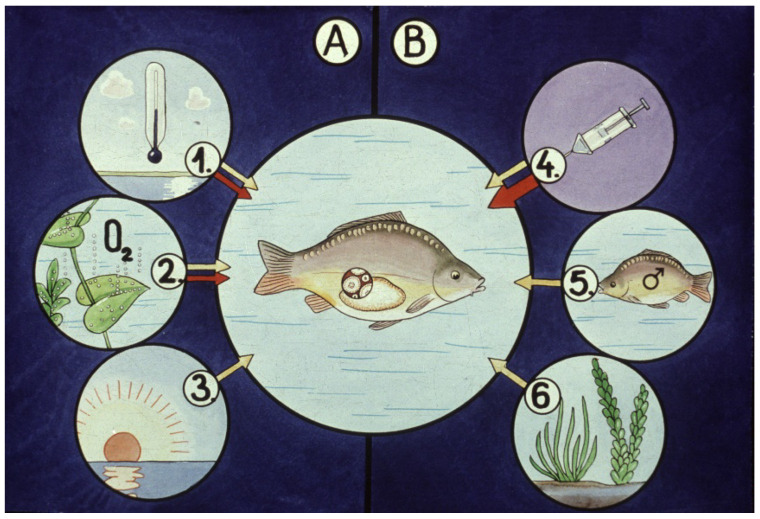
Programmed ovulation in a hatchery. Fish breeders use gonadotropin hormones from external sources. (**A**) Optimal levels of basic factors for normal metabolic processes are the same as for natural spawning (1. temperature, 2. dissolved oxygen, 3. light). (**B**) Instead of natural generating factors (such as point 5 and 6), external gonadotropin from artificially injected hypophysis extract (point 4) induces the ovulation of oocytes [11].

From the dormant stage, the reproductive activity continues when all limiting external factors are optimal for reproduction. These complex conditions are called a “spawning environment” (Figure 10). At the dormant stage of gametes, endocrine (hormonal) activity remains low. Reproductive hormones including gonadotropins like follicle stimulating hormone (FSH) and luteinizing hormone (LH) concentrate in the pituitary gland (hypophysis). By injecting the extraction of collected and conserved pituitary glands into mature fish with gametes in the dormant stage, a programmed (forced) reproduction can be generated (Figure 11).

The basics of the biological pathway of this method were documented by von Ihering, a German-Brazilian zoologist [25]. He was the first to treat ornamental fish with pituitary extract and achieve artificial ovulation.

The next important milestone in artificial fish reproduction was the adaptation of hypophysation in a large *Acipenseridae* in Russia. The establishment of huge water reservoirs in Russian rivers stopped the spawning migration of valuable mature *Acipenseridae* from the Black Sea to the spawning grounds. As a result, high value caviar production started to decrease. A large project started in the 1930s to compensate for effects of the lack of natural reproduction. Gerbilsky and co-workers adapted the Ihering method and elaborated a large-scale protocol, from the collection and storing of pituitary glands, through induced ovulation, to nursing larvae [26]. Thanks to this practical method, these large and protected fish managed to survive the crisis. Financed by the Russian government, millions of young *Acipenseridae* were restocked into rivers, where they migrated to the sea.

Using the Russian method, Jaczo in Hungary propagated sterlet (*Acipenser ruthenus*) in 1953 for restocking into the Danube. The, next year, he successfully used the method to propagate common carp as well. Later, Gerbilsky’s method was adapted to other cultivated fish species, such as common carp.

In the 1960s, when the stickiness of eggs was already resolved and induced breeding (hypophysation) was developed, a complex propagation technique started to be used in fish production. Within years, an efficient common carp breeding protocol adapted to hatchery conditions was developed step by step. Milestones in the development of this method are listed below with some explanations:

## 2. Materials and Methods—Detailed Presentation of Milestones in the Reproduction and Nursing Protocol for Common Carp

The complex and effective method of common carp propagation was summarized in an FAO manual published in 1980 [21]. A few years later, some improved details of the method were reported [27]. To transfer the method to experts in developing countries, other FAO booklets were also prepared [11,28]. Based on these sources and updated with the latest innovative elements, milestones of the method are presented here. Colored figures in the booklets have been inserted with the permission of FAO (Rome).

### 2.1. Broodstock Management

**Figure 12:**
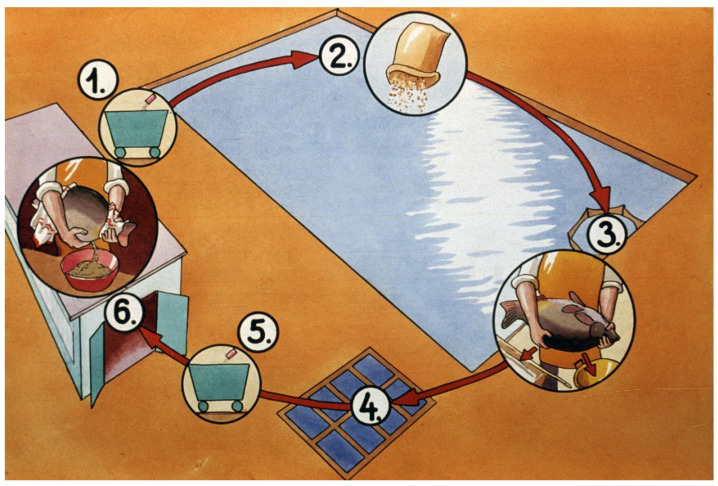
Process of broodstock management during the year. 1. After hatchery propagation, broodfish are transported back to a pond. 2. Intensive feeding on cereals and natural food (planktonic and benthic invertebrates). 3. Autumn fishing from large ponds: after selection, the broodstock is transported to small wintering ponds. 4. Fish feed on protein-rich food in spring to complete oogenesis. 5. Disinfection with quick bathing in salt solution. 6. Ripe fish are propagated in a hatchery [12].

The success of hatchery propagation strongly depends on the year-round feeding management of common carp, which is an omnivorous fish. From a feeding aspect, the year is separated into two parts (Figure 12).

**Figure 13:**
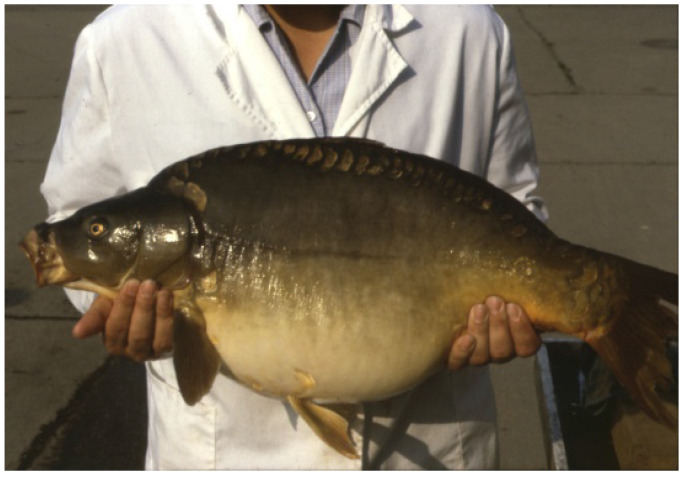
Common carp female with a huge ovary right before propagation (L. Horvath).

Summer period: During reproduction, when releasing a huge number of eggs (around May–June in temperate climate), females lose a lot of energy. A female of 5 kg can produce 1 kg of crude roe, 1/5th of her total body weight (Figure 13). To make up for losses after reproduction, females need extra-rich nutrients. Therefore, in the ponds, they are fed with energy-rich supplementary foods like wheat, barley, and corn. In parallel, additional zooplankton with high protein content are also needed. In the summer period, it is basically impossible to overfeed females, as all of the energy migrates to the ovary and is built into oocytes. The daily amount of grain can reach 3–5% of the estimated body weight of broodstock. As a result of this feeding regime, ovaries of carp females will be regenerated, and by autumn, oogenesis will also be completed. Eggs will reach their final size of 1000 µm in cross section. There is no active egg development in winter.

**Figure 14:**
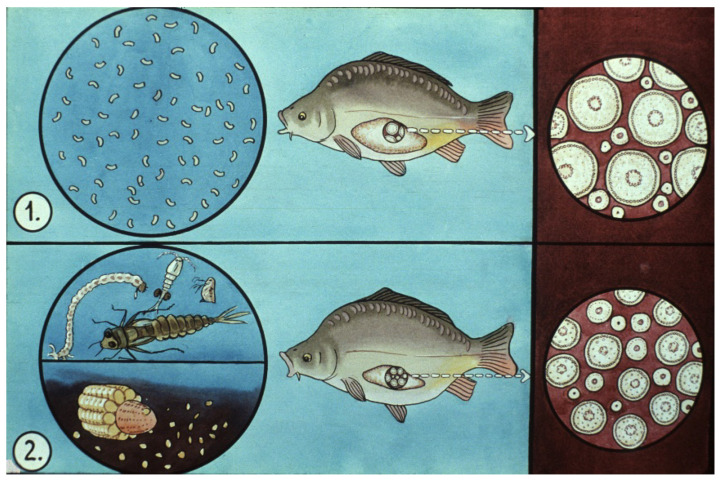
Oocyte stages: 1. From spring until reproduction, and 2. after reproduction, from summer to autumn. Springtime: 1. Maturation of oocytes is completed; they are ready to ovulate (dormant stage). Summertime: 2. Oocytes are growing. By autumn, their development will be completed [11].

In springtime, feeding energy-dense grains (cereals) is prohibited, as it might result in the over-fattening of the ovaries and the liver of females. In early spring, females only need protein-rich food like wholesome pellets with at least 45–50% of digestible protein. Sprouted wheat is effective due to its large amount of vitamin E, also called a “reproductive vitamin” (Figure 14).

**Figure 15:**
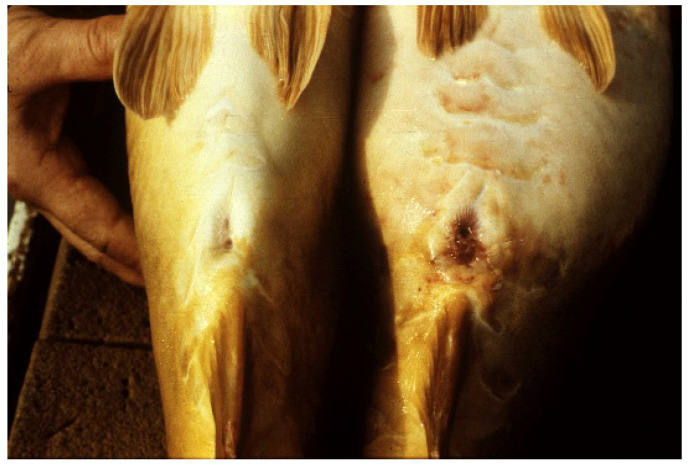
Morphological differences between ripe male and female carp (L. Horvath).

**Figure 16:**
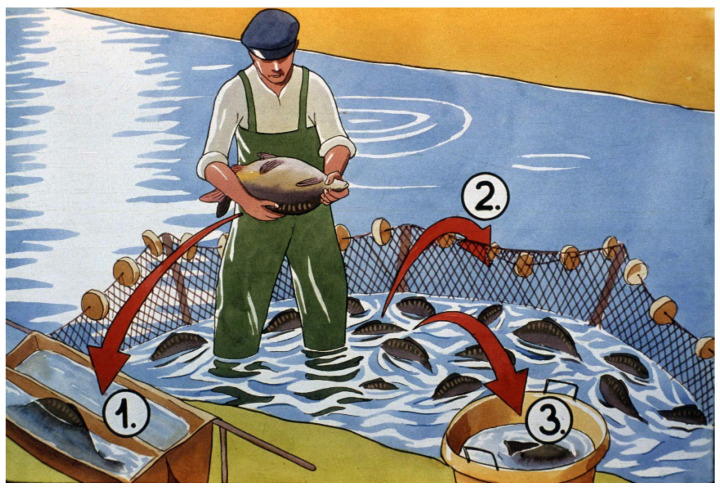
Broodstock selection based on visual signs. Ripe fish are transported to the hatchery (1) and (3). Fish not fully prepared for propagation (2) are taken back to storing pond [11].

In wintertime, the two sexes are kept together in small wintering ponds. To prevent undesirable natural spawning in small bloodstock storing ponds, in spring, when the water temperature reaches 10–12 °C, the mixed population must be separated. By this time, sexual dimorphism is obvious (Figure 15). After sex selection in spring, female carp with eggs in the dormant stage need energy-poor but protein-rich food to prevent dangerous liver fattening (Figure 14). When the water temperature of ponds increases to 18 °C, propagation can be started. First, the smaller females with a soft belly are ready to reproduce (Figure 16). Hatchery activities must be recorded.

#### Anesthesia

**Figure 17:**
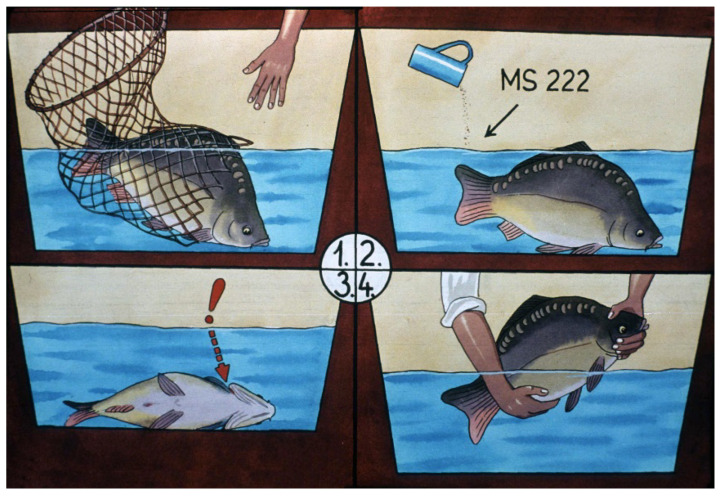
Narcotization process: 1. Netting broodfish and putting them into fresh water with care. This is done with an open-end net to allow the fish to swim out easily, without damaging the scales. 2. Adding clove oil into the tank (1 drop/1 L of water). 3. Monitoring the movement of the operculum (if no movement is visible, the fish has been over-narcotized). 4. Working with narcotized fish is easy (weighing, injecting, stripping, etc.) [11].

**Figure 18:**
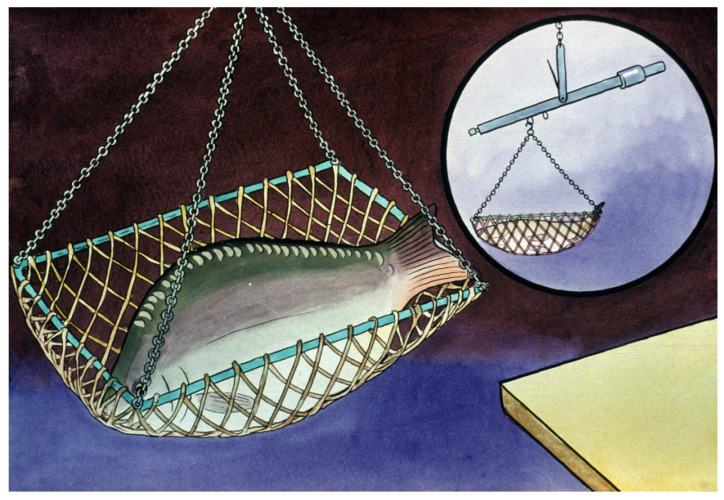
Measuring the weight of broodfish to calculate the required amount of hormone for the treatment [11].

**Figure 19:**
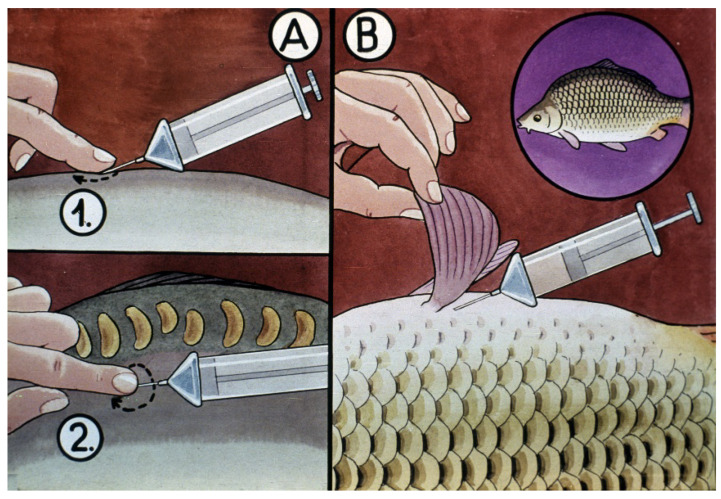
Hormonal treatment. Injecting the hormone solution: (**A**) for mirror carp, solution is injected into the dorsal muscle (1. and 2. to prevent the backflow of the solution, a slight, circular pressing of the index finger should be applied); (**B**) for scaly fish, solution is injected into the body cavity [11].

An important step of the reproduction process is the narcotization of broodfish (Figure 17). In dormant position, fish can be weighed without causing damage (Figure 18), and at the same time, the first hormone treatment can also be carried out (Figure 19). It is best to use a 100–150 L tank for anesthesia. The simplest sedative is clove oil in the amount of 1 drop/1 L of water. In 100 L of water, 6–8 fish can be put to sleep at the same time. It is helpful to aerate the water or supply the narcotizing tank with gaseous oxygen.

Another important step of propagation is the closing of the genital opening of the female fish. The antecedents of this method are told in the following true story:

“When hormonally induced ovulation was still a new method in Hungary, in 1962, fish breeders wanted to demonstrate the programmed stripping of carp eggs to a high-ranking government official (prime minister). When the stripping time arrived, the egg collection had to be started, but the committee was late. Since fish breeders strongly wanted to demonstrate the process to the prime minister, they closed the oviduct of one female for presenting the process. After the arrival of the delegation, this fish was taken out and the roe was removed from it. To the greatest surprise, the eggs remained fertile and the quantity became larger than average”.

**Figure 20:**
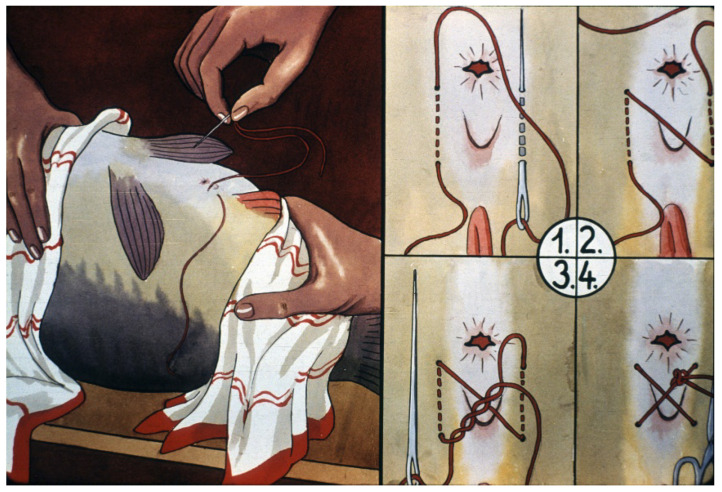
Closing the genital opening to obtain better synchronized ovulation and prevent egg loss (1–4 correct and efficient steps of closing the genital opening) [11].

Afterwards, experiments were organized to investigate the positive roles of closing the genitals of females (Figure 20). Finally, the positive effect on egg volume was statistically proven, and from that time on, it has become a widely followed step of artificial propagation [22].

**Figure 21:**
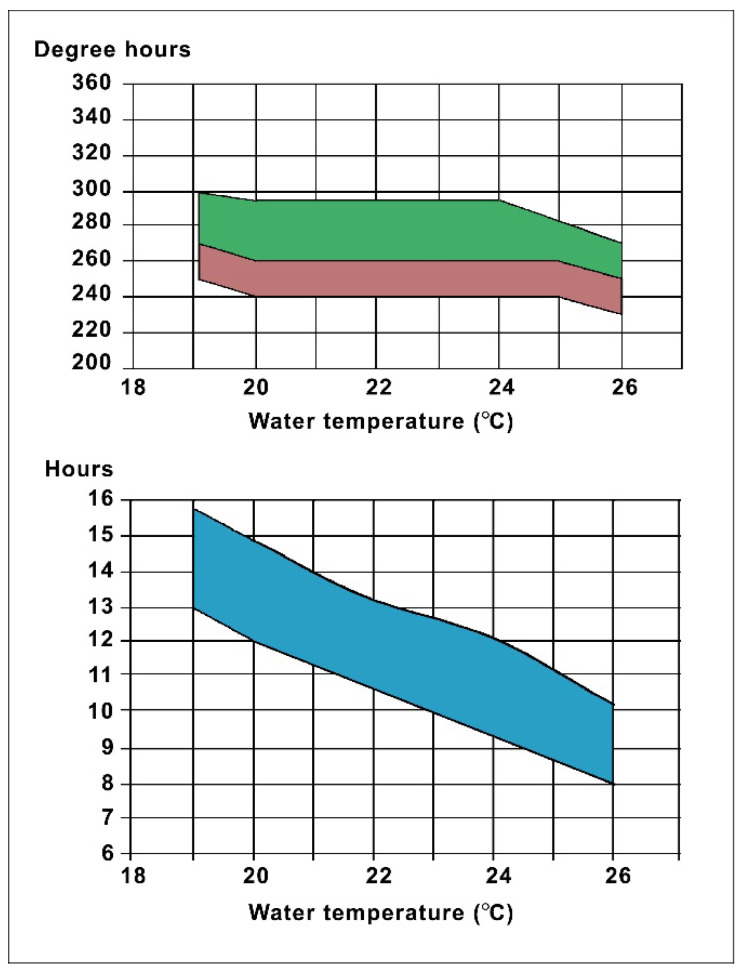
Estimation of time period between hormonal treatment and ovulation at different incubating temperatures. Upper diagram: Connection between heat sum (sum of degrees of every hour within a time interval) and incubation temperature. Green field: heat sum in degree hours necessary for ovulation in case of a one-dose hormone treatment. Orange field: heat sum necessary for ovulation in case of a two-dose treatment. Lower diagram: Connection between the incubation temperature and ovulation time in hours. Degree hours: the temperature measured at every hour and added progressively. Degree days: the average temperature measured in Celsius within a 24-h interval [11].

**Figure 22:**
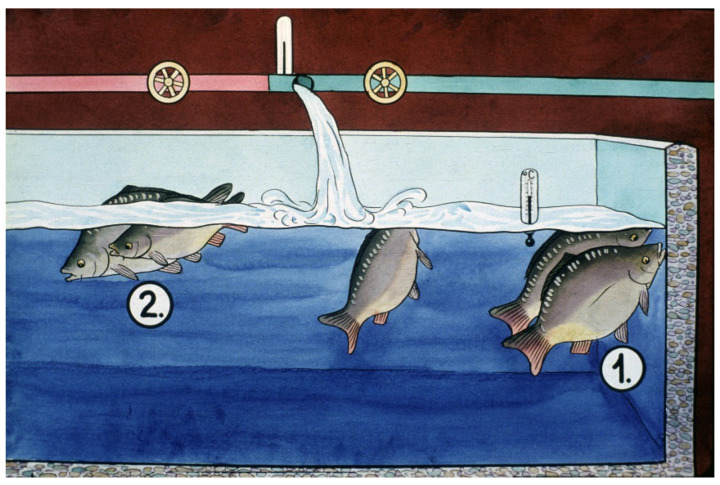
Determination of stripping time by using an indicator male. 1. Carp females swim up to the surface before ovulation to get ready for laying their eggs in the oxygen-rich upper water. 2. The indicator male always starts to spawn with the ripest female with ovulated eggs [11].

There are two ways to determine the optimal stripping time:

1. Measuring the temperature several times during incubation and reading the expected time of ovulation based on the average temperature, as indicated in Figure 21.
2. Using an indicator male (Figure 22). After the hormonal treatment, females and males should be kept in separate basins.

If only a single male is placed between the females as an indicator, when ovulation happens, the male will always start to spawn with the ripest female. If cotton threads with different colors are tied to the dorsal fins of females, it will be easy to select an ovulated fish and only take the eggs from that one. The threads should be attached to the dorsal fin of each fish when the genital ducts are closed, and the second hypophysis treatment is performed.

**Figure 23:**
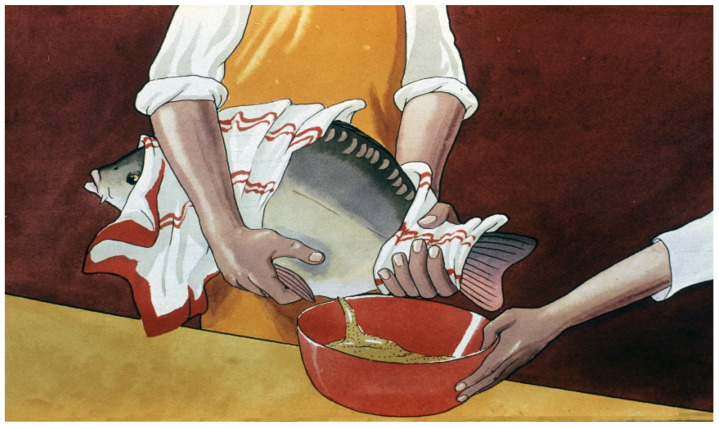
Collection (stripping) of ovulated eggs. It is important to keep the eggs dry while stripping, as in a wet environment, the micropyle of the egg closes, and the egg remains unfertile [11].

**Figure 24:**
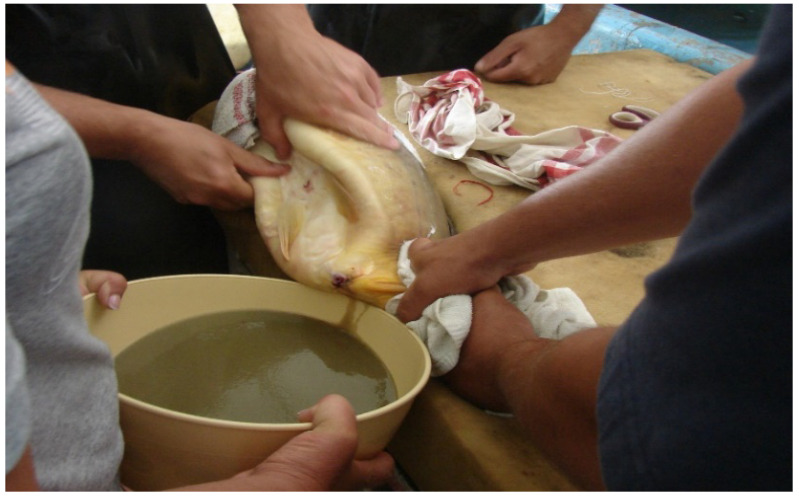
Well-prepared females give large amounts of fresh eggs for hatchery incubation (L. Horvath).

Only females with ovulated eggs should be stripped (Figure 23 and Figure 24); the indicator male will help to select the most mature ones.

In hatcheries, ovulation of females can be induced by hormone administration. As for common carp, different hormone compounds can successfully be applied. Farmers frequently use acetone-dried pituitary gland extracts, or synthetic gonadotropin-releasing hormones combined with dopamine antagonists with high success.

At the beginning of the reproductive season, when not all of the oocytes are in the final stage, and thus in some of them the nucleus is still migrating, to reach a complete ovulation, fish need to have two hypophysis treatments, with 12 h between the treatments. The first dose of hormone treatment results in synchronized oocyte maturation. This dose accounts for only 10% of the total amount calculated.

From the middle of the reproduction period, one dose of hormone treatment results in the total ovulation of carp females. A normally used amount of acetone-dried carp pituitary gland is 3.5–4 mg/kg of female body weight.

**Figure 25:**
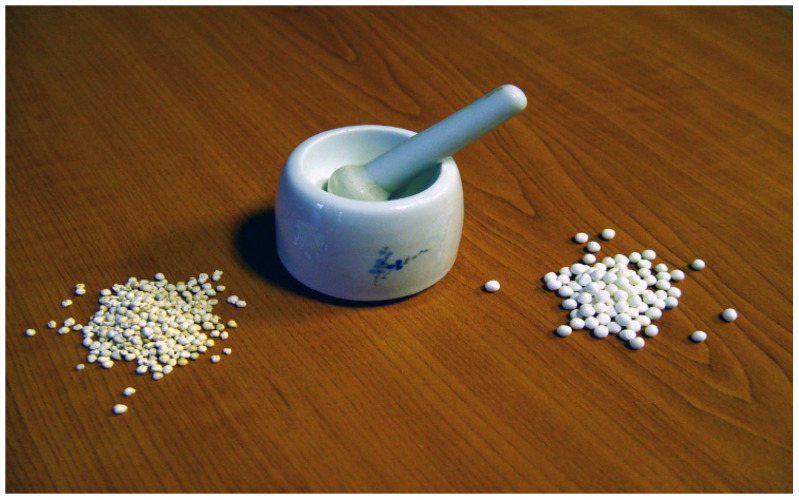
Acetone-dried pituitary glands (**left**), small mortar (**middle**), ball-form pellets of Ovopel (**right**) (B. Csorbai).

From other efficient ovulation-inducing hormone products (e.g., Ovopel), 1 pellet/1 kg body weight should be used (www.ovopel.hu, accessed on 15 May 2023). See Figure 25.

**Figure 26:**
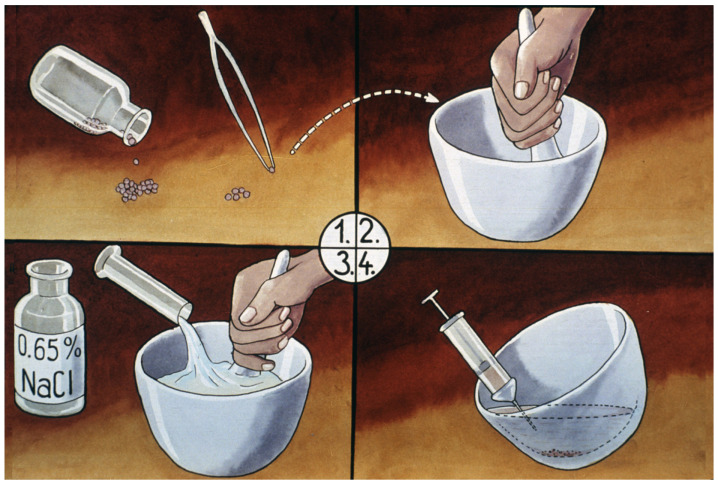
Steps in preparing a hypophysis suspension: 1. The weight of hypophyses is calculated on the basis of the weight of breeders taken into the hatchery. 2. Dried glands are ground into a fine powder in a mortar. 3. This fine powder is then carefully mixed with the calculated volume of physiological solution. 4. The hormone suspension is ready to be injected into the fish [11].

Preparation of a hormone solution (Figure 26): based on the total weight of selected females, farmers can calculate the necessary amount of required hypophysis. A sensitive pharmacy scale is needed to measure the calculated amount of hypophysis plus an additional 10% (as a cover for the losses); then, this lot is minced in a simple mortar.

To every 4 mg of hypophysis 0.5–1 mL physiological salt solution should be added, and then, the solution should be put into syringes. For male carp, half of the number of females should be supplied in a single dose.

Before starting the hormone treatment, it is beneficial to narcotize the fish to prevent jumping and damaging sensitive females with large ovaries. The hormone treatment launches the physiological process of ovulation. This period is very stress-sensitive; therefore, silence is required in the hatchery. Ovulation is a long physiological process regulated by hormones. The final part of oogenesis with the entire ovulation process is explained in detail by Kagawa [12].

The number of fish species involved in pond production is constantly increasing. Traditional methods for ovulation induction, e.g., hypophysation or treatment with human chorionic gonadotropin, do not necessarily lead to the expected results for all species. Therefore, it would be justified to explore the fundamental physiological processes preceding ovulation. The most critical stage just prior to ovulation is known as final oocyte maturation, which is characterized primarily by the breakdown of the oocyte nuclear envelope. This process is under endocrinological control and is marked by the production of different gonadal steroids. Investigation into the nature of these mediators in different fish species with economic importance would be a key element conducive to reaching higher productivity.

When ovulation is completed, the eggs become free from the ovary septum. To accomplish this long biochemical process, an optimal incubation temperature and heat sum are required (see Figure 21). By monitoring the real incubation temperature, the potential stripping time can be estimated. There is also a significant difference in the ovulation time depending on the number of injections. For common carp, freshly ovulated eggs can be manually stripped from the ovary.

**Figure 27:**
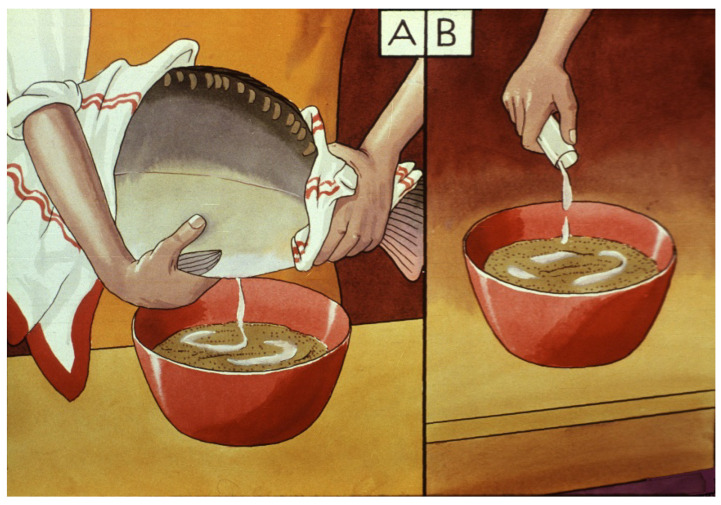
Sperm stripping: (**A**) directly on the eggs. (**B**) First, the sperm is collected in a small jar, then mixed with the eggs [11].

**Figure 28:**
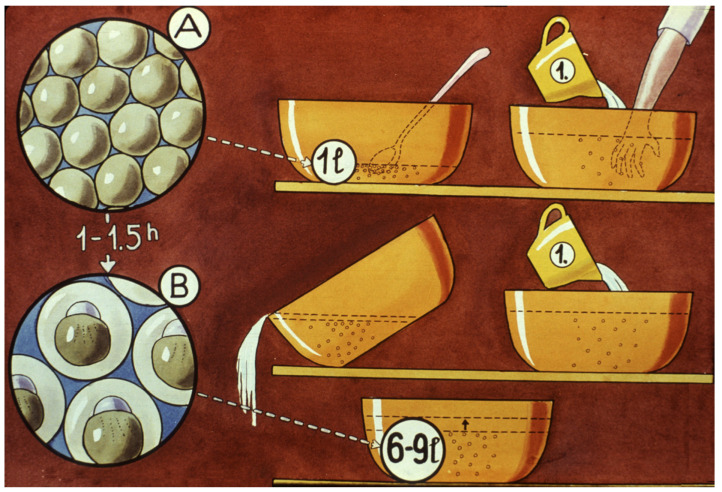
The swelling process of fertilized carp eggs. (**A**) The shape of eggs immediately after fertilization. (**B**) An hour later, swollen carp eggs with larger perivitelline space. During the swelling process, the mass of eggs is washed through with the solution containing salt and urea [11].

Carp sperm collection is a simpler process. Sperm can be stripped into different small cups or directly onto the mass of eggs (Figure 27). One liter of eggs is fertilized by 5–10 mL of freshly gathered milt. As suggested by Vransky (1856) in the method of dry fertilization [14,15,16,17,18], after mixing the two types of gametes (eggs and sperm) in a dry environment, about 50–100 mL of fertilizer solution is added and mixed with the gametes. According to Woynarovich [24], the original fertilizer solution includes 40 g of table salt and 30 g of urea in 10 L of fresh water. The temperature of the solution should be the same as that of the egg–sperm mixture. Continuous stirring is necessary to prevent the adhesion of eggs. When the eggs start to swell, 20–30 mL of fertilizer solution must be gradually added in small portions. The swelling process lasts for about an hour. By the end of the procedure, the mass of eggs expands about 6–9 times in volume, due to the development of a large perivitelline space around the ovum, which is full of fluid (Figure 28).

At the end of the swelling process, a tannic acid treatment is used to remove the stickiness of eggs. When 3–5 g tannic acid is added to 10 L of clean water, it will form a protein coagulant solution, which eliminates adhesive compounds from the egg surface. About 1 L of tannin solution is used to treat 4–5 L of swollen eggs. As soon as the solution is added, eggs should be gently but quickly stirred and then diluted with 5–10 L of clean, well-aerated hatchery water. After eggs have settled, the diluted solution should be poured off. The entire procedure should be repeated 2–3 times, and then the eggs can be transferred into the Zug jars. After the tannic acid treatment, eggs will float separately in vertical incubators (Zug jars), as carp eggs will change from substratophile to pelagic form.

From fertilization, eggs need around 3 days at 20 °C to hatch, which is about 60–70 day degrees.

**Figure 29:**
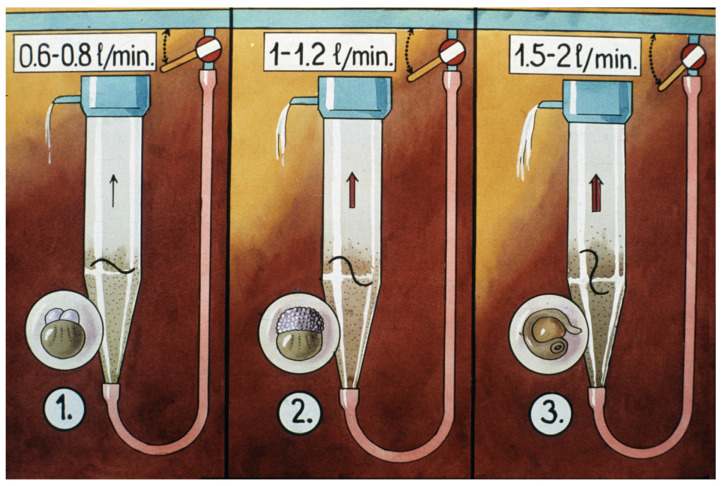
During incubation, the water flow should be adjusted to the actual development stage of the eggs. 1. For the first 10 h, about 0.6–0.8 L/min. of water should pass through each jar. 2. When the blastula stage starts, the water flow should be increased to 1–1.2 L/min. 3. When the tail, the eyes and the pigmentation of the embryos become visible, the water flow should be increased to 1.5–2 L/min. Arrows indicate the way and strength of the water flow at different stages of egg development [11].

In a 7 L Zug jar, 2–3 L of swollen eggs can be incubated. The water for egg incubation comes from an equalizing tank, as eggs require a stable water flow, which should be 0.5–1.2 L of water/minute, depending on the status of the eggs (Figure 29). Incubation water transports the necessary dissolved oxygen and washes the wastes deriving from egg metabolism.

**Figure 30:**
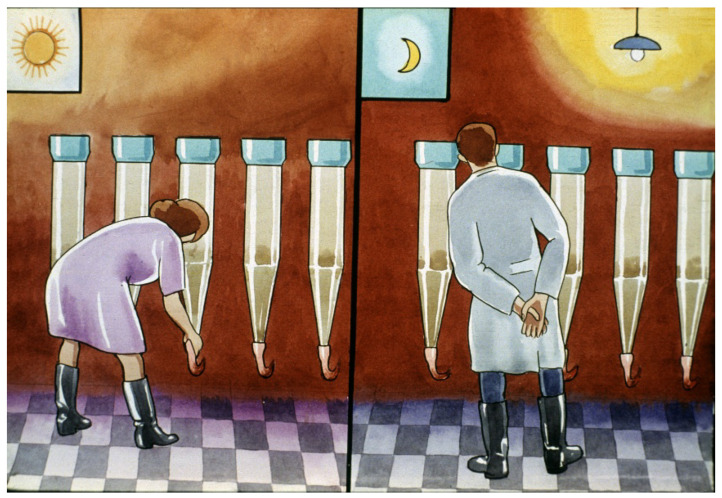
Continuous monitoring of water flow-through on eggs and regulation of water amounts [11].

Without this water flow-through, eggs would quickly be damaged and would die within a short time, so continuous monitoring in every hatchery is inevitable (Figure 30).

**Figure 31:**
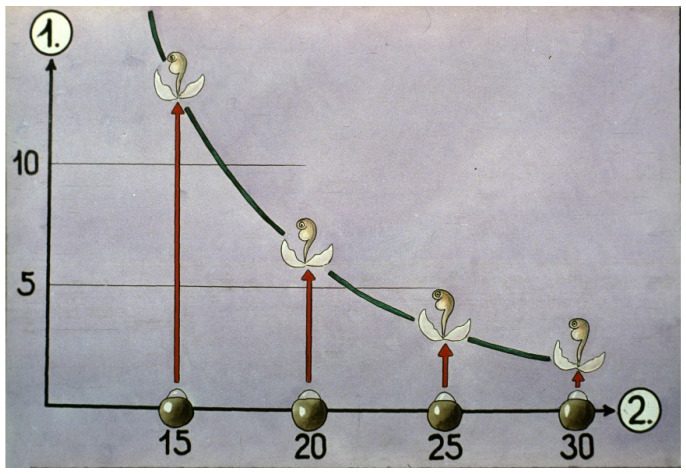
Connection between (1) hatching days and (2) incubation temperatures [11].

Water temperature has a significant effect on hatching: the higher the water temperature is, the quicker the eggs go through their development, and the less time the procedure needs until hatching (Figure 31). 

**Figure 32:**
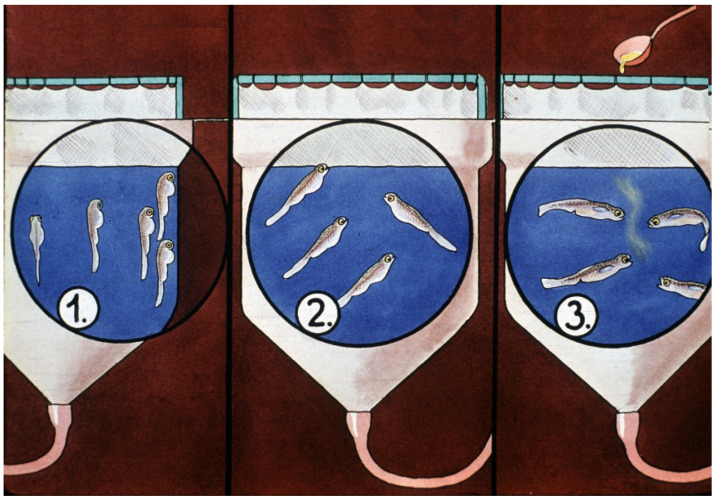
Carp larvae in large Zug jars. 1. After hatching, larvae hang on the wall of the containers. 2. Later, on the second day, larvae start to swim up to the water surface. 3. After filling up their swim bladders with air, they are ready to swim horizontally as well and start eating. The traditional first food in hatcheries is a fine suspension of hard-boiled chicken eggs [11].

After the start of hatching, carp larvae are transferred to large 200-L Zug jars from the 7-L ones (Figure 32). These large plastic containers have a fine net ring around the top to prevent the escape of fish larvae.

Larvae stay for 3–4 days in such jars. When they start their exogenous feeding, a fine suspension of hard-boiled chicken eggs is provided as starter food. Within a short time, the young carps will be transferred to well-prepared nursing ponds.

## 3. Carp Fry Rearing, Focusing on the Adequate Size of Natural Food (Zooplankton)

**Figure 33:**
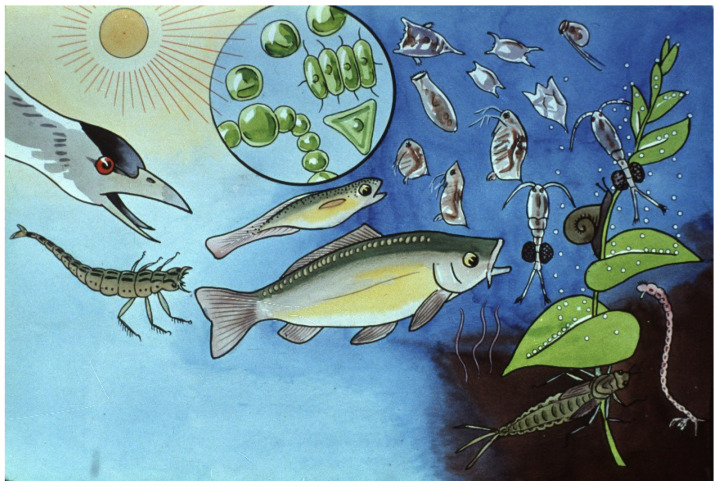
In pond conditions, young fish, their food organisms and predators live together in the same habitat. Many predators (water birds, insects and their larvae, frogs and even some groups of zooplankton, e.g., Copepods) hunt on young carp. It is the task of fish breeders to protect young carp from their enemies. Another important task is to maintain adequate natural food for quickly growing fish larvae. Small-size fish need small-size zooplankton, and as they grow, the size and amount of their food must grow as well [28].

Nursing ponds are natural habitats with interacting organisms living together (Figure 33) in a specific ecosystem.

### Living Organisms in Nursing Ponds

**Figure 34:**
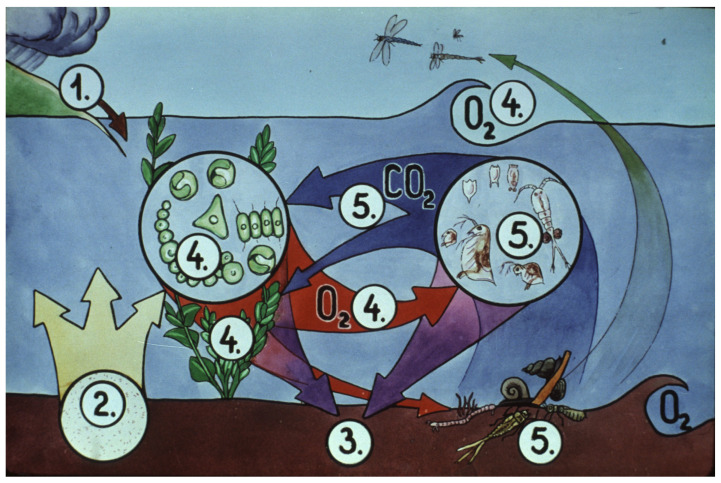
There are several factors in the biological cycle that influence results: 1. Soil erosion washes nutrients into the water, and bacterial activity in the water column and at the bottom of the pond releases additional ones. 2. and 3. Organic matter of bottom mud, which consists of thousands of dead organisms, is partly recycled into the production system in this way. 4. Oxygen in the water originates mostly from plant photosynthesis and absorption from the atmosphere. 5. Carbon dioxide (CO_2_) is the result of respiration of animals. This is utilized by phytoplankton and other water plants [28].

The most important members of pond ecosystems likely to live in nursing ponds in the temperate zone of Europe are shown in Figure 34.

The composition of a pond ecosystem is a determining factor for the survival and production of young carp. Tiny water plants such as algae are important primary producers, synthetizing organic compounds and providing the bases of the food chain. Some other species of the ecosystem play a role as important food organisms, like, e.g., rotifers as starter food and Cladocera and young Copepods together with larvae of mosquitos as mass sources of proteins and fatty acids.

Predators of young fish and different water insects, including their larvae, can cause high losses in young fish populations. Fish breeders utilize natural sources of pond ecosystems (through food organisms) and protect young fish from predators. Besides food of natural origin, breeders also provide supplementary feeds (fine-ground cereals, as natural food is not able to provide enough energy for rapidly growing fish stocks).

## 4. Breeding Process from Hatchery to Market-Size Carp

**Figure 35:**
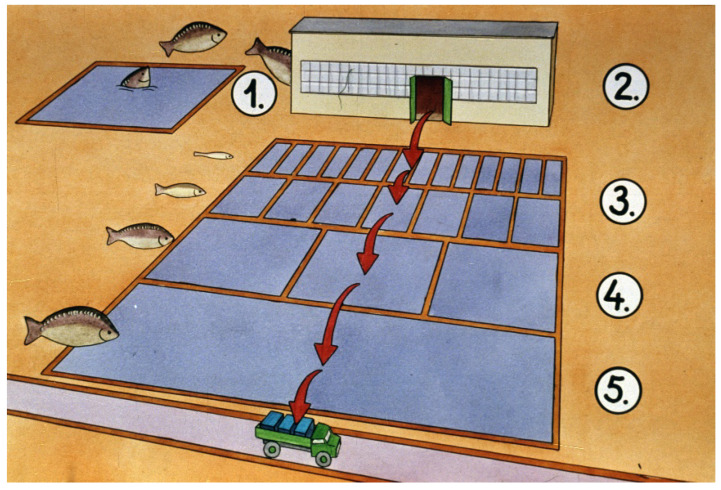
In traditional pond fish production, carp fries reach market size within three production seasons. 1–3. First production season. 1. The broodstock is kept separately to produce good-quality eggs and sperm. 2. Carp fry stocks are nursed in well-protected (small) and specifically prepared nursing ponds for about 3–4 weeks. 3. At the end of the nursing period, advanced fry is transferred to larger ponds called fingerling ponds. 4. In the second production season, fingerlings are raised in lower densities and in larger ponds to achieve quick growth. 5. Carp breeding for markets happens in the third season [28].

In the temperate zone in a traditional pond culture, carp need three seasons to reach market size (Figure 35). A basic rule in carp breeding is that small fish are placed into small, well-protected ponds, while larger fish are stocked and reared in larger ponds.

**Figure 36:**
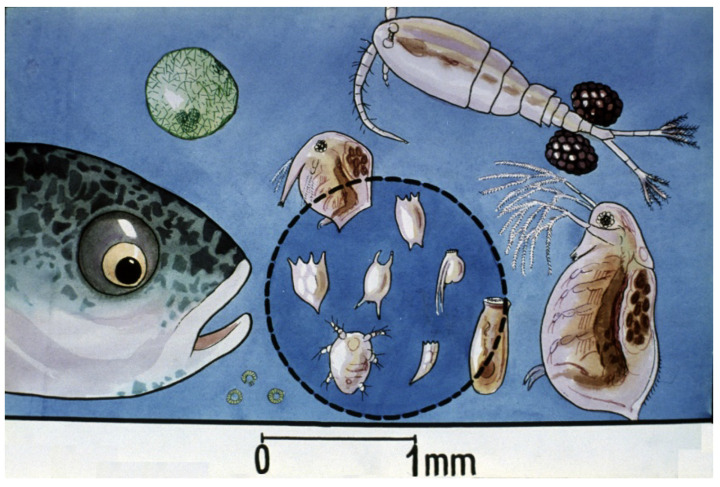
Mixed zooplankton with 3–4-day-old carp fry in a nursing pond. Slowly moving rotifers (within the blue circle) are optimal as starter food for young carp [28].

In the current review, only the nursing phase is investigated and presented. The pond breeding process starts with the stocking of swim-up or feeding larvae fry coming from a hatchery (Figure 36). They are introduced into specifically prepared nursing ponds. The development of the protocol started in 1957, when the first publication proved that the so-called nursing period, which is a 3–4-week-long period after fry are stocked into earthen ponds, results in better survival and production than that of a whole-season-long (one-phase) breeding system.

This new rearing strategy is called a “two-phase rearing method”. The presently applied nursing method has been used for 50 years. When a complex hatchery method of carp reproduction was first applied, it resulted in a large number of young carp. When such produced fish were introduced into earthen ponds, unfortunately, their survival rate was very low.

**Figure 37:**
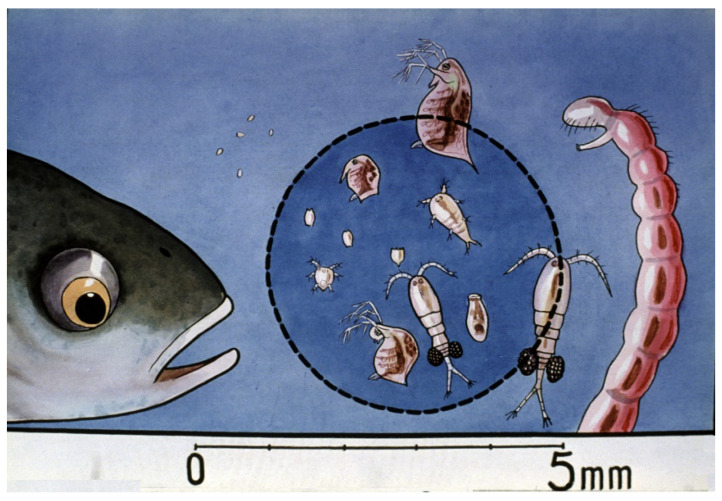
Relationship between two-week-old carp and mixed zooplankton. Fast-growing young carp can predate on cladocerans and even on copepods [28].

Comparing survival results of carp fry of “natural” (Dubits method) propagation to that in hatcheries, survival rates showed much lower levels. The phenomenon was intensively investigated, and within a few years, the underlying reasons were clarified. When “mosquito carp” from Dubits spawning ponds were fished and transferred into other rearing ponds, the size of the young carp was 10–12 mm. These fish had already adapted to harsh pond conditions, learned to catch food organisms, and the size of their mouth was large enough to predate on smaller cladocerans and even on adult copepods (Figure 37).

Contrarily, carp of hatchery origin (with only 6–7 mm of body length) transferred directly from protected hatchery conditions had no practice in catching fast-moving live food, as in larvae containers, they were only fed with suspended, hard-boiled chicken eggs. This small difference between the two groups meant an important advantage for young carp of pond origin. The largest fries from spawning ponds were saved from predation by copepods.

Detailed laboratory and field investigations on zooplankton stocks proved the harmful effect of tiny crustacean planktonic organisms (carnivorous copepods). Their predation caused low survival rates of carp fry. To understand how copepods can become dominant among the zooplankton and cause remarkable losses in young fish populations, a gradual modification of different zooplankton groups, called plankton succession, provides an explanation.

### 4.1. Zooplankton Succession in Nursing Ponds

With the long-term investigation of changes in zooplankton groups in freshly inundated nursing ponds, the complicated connections between different zooplankton groups were recognized [29].

After filling the pond, first, the smaller and parthenogenetically faster-reproducing group, the rotifers, will be the dominant ones within a mixed zooplankton population. Within days, Cladocera groups of different sizes will follow rotifers, while over a longer period, predatory copepods will become the predominant group. This process is called zooplankton succession.

From the results, it was evident that the optimal time of stocking young fish is after the inundation of the pond, when rotifers are in a reproduction phase. Unfortunately, at this early stage, the density of rotifers is still low, and only limited amounts are available for fish to grow on.

Researchers have also recognized the contradiction that even though slowly moving, small-size rotifers would be an optimal starter food for young carp of hatchery origin, their density is low even at the time of pond inundation; moreover, later on, dangerous copepods will predominate [29], which leads to the low survival rates of fish fry. By examining the structure of zooplankton in the rearing ponds, it was found that poor survival was due to unfavorable compositions of zooplankton populations. Dense populations of copepods that existed in pond water had a strong influence on the survival of fish fry.

**Figure 38:**
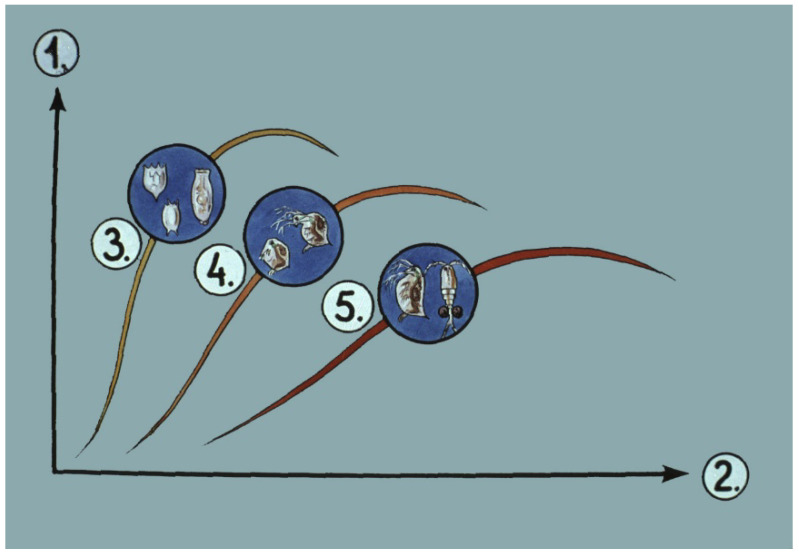
Tendencies of the three important zooplankton groups in nursing ponds. 1. Amounts of zooplankton groups. 2. Stages of the nursing period (3–4 weeks in total). 3. Quickly reproducing small-size rotifers. 4. Small-size cladocerans. 5. Large-size cladocerans (*Daphnia* sp.) and predatory copepods [28].

If early fries are introduced to nursing ponds when copepods are dominant (Figure 38—5), only a few fish will be able to survive due to the attacks by predatory zooplankton.

### 4.2. Zooplankton Selection

Fortunately, fish researchers have managed to develop two efficient methods to increase fish survival.

One of them is a selective chemical treatment of nursing ponds before introducing fish fry.

Using organic phosphoric acid esters in a concentration of 1 ppm for zooplankton selection results in the elimination of all crustaceans without damaging the rotifer population. In a nutrient-rich environment without predators (copepods not only attack young fish but small rotifers, too), rotifers are able to reproduce intensively and form a large population within 2–3 days [30].

In recent years, access to organophosphoric acid esters, which were previously successfully used in aquaculture, has become more and more difficult. Therefore, the effectiveness of plankton selection, which plays a key role in advanced fry rearing of Cyprinids, has decreased significantly. The inorganic compound calcium hypochlorite could potentially be used instead of organophosphoric acid esters. To take advantage of the broad benefits of calcium hypochlorite, extensive laboratory and field studies of its effect on phytoplankton and zooplankton are recommended.

**Figure 39:**
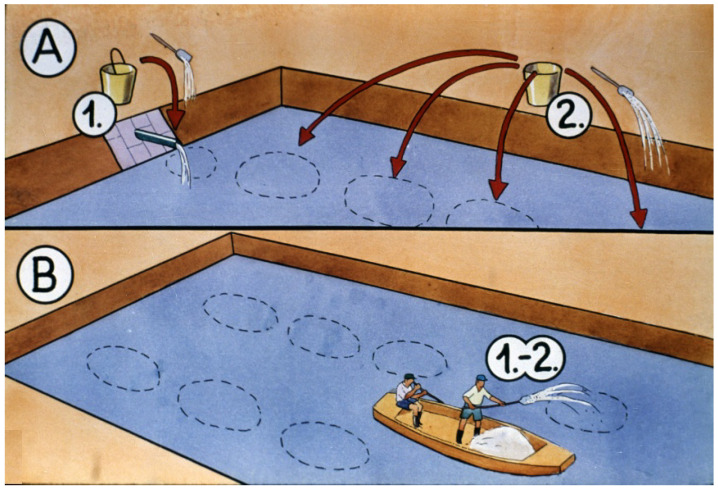
Chemical treatment of a nursing pond. (**A**) 1. Powder or liquid forms of chemicals are dissolved in water 2. This solution is distributed in different parts of the nursing pond from the embankment. (**B**) In larger ponds, the same treatment is provided from a boat [28].

**Figure 40:**
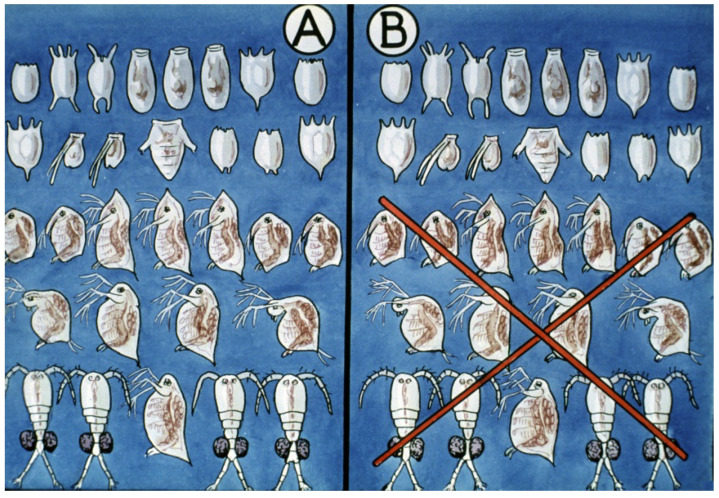
Structure of zooplankton stocks: (**A**) In non-treated nursing ponds. (**B**) After a chemical treatment, only rotifers stay alive, resulting in an optimal structure of starter food for young fish [28].

A strong (5 ppm) chlorinated chalk treatment (Figure 39) can also be efficient to develop an expected structure of the zooplankton. This chemical kills all planktonic organisms and even trash fish species like *Pseudorasbora parva* or *Carassius auratus*, but loses its strength within 24 h. If fish producers introduce a small amount of live collected plankton from another pond, quickly reproducing rotifers will develop a large population within 2–3 days (Figure 40). These two types of treatments are able to significantly increase survival rates of the progeny [31].

**Figure 41:**
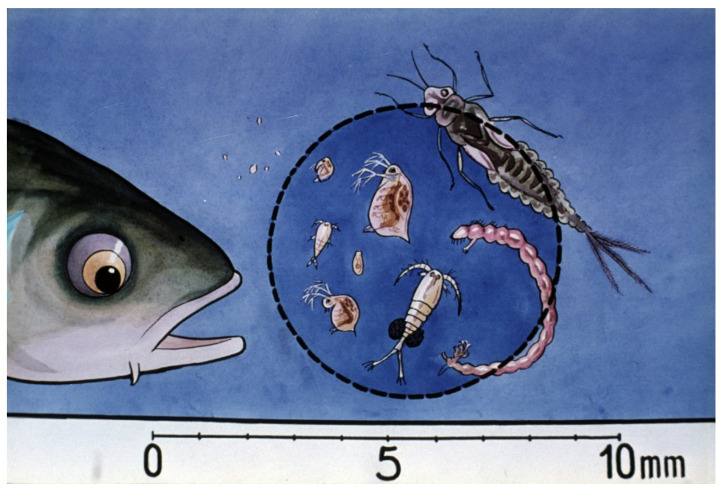
By the end of the nursing phase, young carp are able to consume all kinds of food organisms in nursing ponds, even large chironomids (red worm) and young insect larvae [28].

In the last 5–10 days, fry feed on different planktonic crustaceans. They are also able to consume small larvae of insects such as chironomids and mayflies (Figure 41).

With high stocking densities, young fish not only require natural (planktonic) food but daily supplementary feeds as well. Good-quality artificial feeds like fine soybean powder, and later also ground soya and cereals in increasing amounts, all serve better survival and quick growth rates (from 1 L of fine powder to 5–6 L of ground food for a hundred thousand fish) [27].

## 5. Results—General Evaluations of Data Collected during Application of the European Hatchery and Early Nursing Method

### 5.1. General Outlook

In the last the 50–60 years, the above presented, well-elaborated propagation and nursing method has been used in many central European fish farms. In particular, many fish farms used it in the Carpathian basin, where many important parts of the method were elaborated and developed.

The whole complex method was applied and tested not only on a small scale but also on a large scale by fish farms. Published data on the results are very limited, because of the difficulty involved in performing all of the precise steps and at the same time documenting the measured data in booklets. These double tasks need high expertise, patience, and talent from fish breeders. The objective biological and statistical evaluation of the results is also difficult, because analysis requires extensive recorded data for each important section of the whole process and, in the case of embryo- and larva-genesis, a practical knowledge of the field of microscopic investigation is necessary. These difficulties explain the very limited numbers of publications in this field [29]. For evaluation, it seems appropriate to analyze different biological stages within the whole method (such as broodfish, embryogenesis, and nursing).

### 5.2. Data Collected from Carp Bloodstock Reproduction

Some important data are shown in Table 1, providing important information to evaluate the effectiveness of broodfish reproduction.

### 5.3. Data Evaluation Regarding the Brood Stock

The quality of matured females serves as the basis for the whole hatchery propagation process, so it is important to evaluate their biological status and their reaction to the propagational interventions. Before the evaluation of the quality of females prepared for reproduction, we must start with a discussion of the methods, under which one can estimate the reproductive potency of female carp. In order to estimate the reproductive capacity of the female carp, it is necessary to measure the ovaries, which are full of mature eggs. An important index can be used to determine the reproductive capacity, the fecundity of females. The gonadosomatic index (GSI) shows how the size of the ovary that stores the eggs is proportional to the total body mass of the female. The formula for this GSI is weight of the total ovary ×100/total weight of the female [29].

The specially prepared brood stock (females) provides the initial starting point for the calculation of the next fingerling generation necessary for fish farms in the next season. For the estimation, determination of the potential number of maturing oocyte is needed.

According to Huet (1986) 1 kg bodyweight of matured carp can contain 100,000 oocytes [34]. With this, data breeders can calculate the biological potency of the carp females. When fish reach the reproductive season (in the case of carp, this is May–June) the largest part of the ovary contains mature oocytes, and the rest contains the underdeveloped young oocytes and layer of ovary. At this time, the GSI practically shows the whole number of ripe oocytes, so from the weight of the ovary present, the amount of matured oocytes can be calculated. In matured carp females, the weight of the ovary is about 20% of the total body weight. The GSI data can help with the estimation.

There is another important reproductive index named the pseudo-gonadosomatic index (PGSI). It measures the weight of the stripped egg mass in grams/weight of fish before stripping, in grams × 100 [35].

In the early phase of the hatchery method application, one of the first established large-scale hatcheries in Hungary (Dinnyés in central Hungary) had an excellent practical leader named George Jonas, who was able to collect highly precise data on treated female carps. The late “old uncle George” recorded annual data on hatchery reproduction of common carp over a period of 17 years (1980–1997) [29,36].

The number of hormonally induced females in this long period totaled 2620 female carp, of which 2086 responded with positive ovulation to the hormonal treatment (hypophysation). This means 80% successful ovulation, which is a very impressive result. (According to earlier data from other authors [11], the successful ovulation ratio had a larger range, between 60–90%). According to the summarized data for the 2086 carp females, at the time of spring reproduction, ovulated eggs (PGSI) accounted for more than 20% of the total body weight [29].

Analyzing the data from the mentioned 17-year period, if females were selected in three different groups by weight (small 4–5 kg, medium 5–7 kg and larger than 7 kg size), it can be statistically shown that the females in the smallest group but ripe with a soft belly were the first to be ready for ovulation. At the beginning of the season, the ratio of smaller, soft-belly females was higher than that of the larger-sized fish [29,36].

### 5.4. Data Collected from Hatchery Actions

In Table 2 lists detailed results of hatchery propagation including embryogenesis.

### 5.5. Evaluation of Data on Hatchery Procedures

Data that characterize the hatchery procedures are presented in Table 2. Common carp belongs to the group of warm-water fish; therefore, the optimal temperature for ovulation and early development of carp eggs is about 20–23 °C.

To induce programmed ovulation, hormonal induction is needed in hatchery conditions. (Contrarily, during natural spawning, ovulation is induced by some environmental factors, such as flooding, increased spawning temperature (8–20 °C), weather changes, etc. [23,29]).

According to the practical experiences after hypophysation, matured females produce crude eggs (ovum) equivalent to 20% of their total body weight (PGSI). One kilogram of freshly ovulated eggs contains, on average, 800,000 eggs ready for fertilization [29,37].

The most effective fertilizing solution contains 40 g of common salt and 30 g of urea in every 10 L water. The swelling procedure lasts 1–1.5 h. During the swelling treatment, the volume of the crude eggs increases up to 3–6 times [24]. One liter of swollen eggs contain 120,000 egg, of which 90–95% are fertile. A 60–70 day-grade is needed for total embryogenesis [31,32]. Following the swelling process, eggs are transferred into Zug jars, 1–2 L in every vessel. After 3–4 days, larvae are hatched in a very large proportion (more than 95%). The period of the non-feeding larval stage lasts 3–4 days [31,32]. 

At the end of the larval stage, young carps are ready to feed. Their first food is suspended hard boiled chicken eggs. When larvae have good practice in feeding (from half a day to one full day after the first feeding), they are ready for pond nursing and transfer to a well-prepared open pond.

### 5.6. Data Collected from Nursing of Young Carp (Fry) Living Is Open Ponds

Table 3 contains practical data on the nursing process. 

### 5.7. Evaluation of Pond Nursing Data

Nursing periods last a relatively short time, only 3–4 weeks. During this period, the high-density stocked fish population (600–1000 individuals/m^2^) consumes all of the food organisms and starts starving. Therefore, their immunity decreases quickly, and within a few days, the survival rate of the young carp declines; therefore, after 3 weeks, fishing of the nursing ponds must start.

At that time, the size of the carp is only 2.5–3 cm, and their weight is 200–300 mg; therefore, the fishing operation requires high care from fishermen.

Their survival mainly depends on the amount and size of living food organisms existing in the form of pond zooplankton. At the time young carp start to feed, their livers and stomachs do not produce enough digestive enzymes, and the digesting activities have low efficiency. In the pond ecosystem, only the tiny members of zooplankton are good feeding sources, but in general, the mixed population of zooplankton contains few adequately sized members. Then, the main task of fish breeders is to manage the pond zooplankton, among which the small and slowly moving rotifers create a dense population. The early zooplankton selection solves the problem of the presence of high amounts of starter food organisms after inundation and manuring of the nursing pond. A quick and effective chemical treatment kills the dangerous, larger predator members (copepods) of the zooplankton population and helps the fast-reproducing small rotifers and smaller cladocerans create a dense population. Additionally, from the time they are released into the nursing ponds, young carp require supplementary feeding for quick growth and good survival.

## 6. Discussion of European Carp Propagation

The carp propagation method popular in Europe has some advantages and disadvantages. In general, among the most important advantages, fish farmers can readily calculate their needs in terms of biological power sources, physical infrastructure, and economic background. In the case of accurate registration of subprocesses data, the most important advantage is that one can determine the number of mature carp females required to meet the annual demand for a one-year-old carp population in the next season. One can calculate the water requirements of the hatchery, the hormone/pituitary needs, the area of the necessary nursing ponds, the feed requirements of the nursing fries, etc.

Summarizing the needs of all subprocesses, the total annual costs required for operation can be estimated with high confidence. The disadvantages are the need for continuously increased biological knowledge of fish reproduction and pond ecosystems, heavy investments in skilled human labor, practice in basic microscopy techniques, and relatively advanced technical infrastructure (special equipment, continuous power supply, etc.).

Overall, the advantages outweigh the disadvantages, and it can be stated that the complex method has high effectiveness. This can be illustrated with a simple example. As a starting point, take only one mature female carp. Based on the data in Table 1, Table 2 and Table 3, presented in the previous chapter, in the case of good preparation of females and successful hormone induction, 1 kg of fertile eggs (800,000 eggs) will be the result, which translates into a PGSI of 20% in the case of a large, 5 kg female. This egg mass is 90% fertile, meaning that 720,000 of the carp embryos (800,000 × 0.9) are alive. The total loss during incubation is 10% (720,000 × 0.9 = 650,000). The larvae losses in the hatching and larval period are another 10% (650,000 × 0.90 = 580,000) [27,32]. In the nursing period, the survival rate of larvae in the pond is only 30% [33,37], in this example leaving a total of 174,000 advanced fries. (When exogenic feeding of fries starts in a new nursing condition—from a hatchery to a pond—significant losses can be detected because of the huge difference between the well-protected hatchery condition and the less-protected natural-like pond condition. If the survival rate of young carp in this phase can reach 30%, breeders will be satisfied.

According to Halver et al. (1984), from advanced fries to one-season-old age, the survival rate in a pond is about 70% [27], so, following the above example, 174,000 × 0.7= 120,000 one-season-old fingerlings will be produced from only one female (but only if all of the effective factors work at the optimal level). In this case, the survival from ovulated eggs to one-season-old age is 15%.

Conversely, according to Szuvorov, in nature, the survival rate of the same one-season-old freshwater fish is only 0.01% [35]. As a comparison, in nature, from the same number of eggs (800,000), from freshly ovulated ovum to the end of the first season, the above survival rate will result in only 80 one-season-old fish. The difference is incredible.

When investigating other existing propagation methods, comparison is very difficult. In the natural-like spawning methods, which are very popular everywhere in Europe, South America and Asia, the production of one-season-old carp is larger than that occurring in nature, because the fish breeders try to protect their young fish; however, if the fish breeder has no information and no data from subsections of reproduction, or no data registered on the important biological phases (ovulation success, embryogenesis, survival of fries) the pre-estimation of future yearling production is impossible. From this short description, it is clear: plannability and reproducibility are important advantages of common carp propagation in a hatchery.

## 7. Conclusions

The common carp propagation in hatcheries and fry nursing method in specially prepared ponds are very effective fish breeding processes that offer large numbers of seed stock (advanced fries) for either intensive growth in RAS systems or other intensive breeding methods. Their product, 3–4-week-old young carp, can be stocked not only in the intensive systems but in semi-intensive pond breeding production as well. As well as increasing the intensity in animal breeding, it poses fewer environmental hazards [1], protects nature and decreases the impact of global warming.

From a breeding perspective, the main advantages of the hatchery method of carp reproduction are its reproducibility, plannability and safety, in addition to high productivity. Therefore, in the future, it can be predicted that this method will become more and more popular and spread to more fish farms across different continents.

## Figures and Tables

**Table 1:** Data on reproductive biology of common carp females in temperate zones. Data collected from [27,32,33].

Specification			Unit	Common Carp
Maturation	Age	Male	Years	2–3
		Female	Years	4–5
	Weight	Male	kg	3–4
		Female	kg	4–5
	Length	Male	cm	25–30
		Female	cm	30–40
	Spawning	Month		IV–VI
Water temperature			°C	16–22
Number of eggs gained from 1 kg of weight of				
females			Thousands	100–200
Number of eggs gained from a female			Thousands	200–1500
Diameter of eggs				
Dry			mm	1.0–1.5
Swollen			mm	2.0–2.5
Number of eggs in 1 kg				
Dry			Thousands	700–1000
Swollen			Thousands	80–120
Time between fertilization and hatching			Daygrade	60–70
(at optimum incubation temperatures)			Days	3–4
Fertilization rate			percentage	80–95
Hatching rate			percentage	90
Survival in 200 L vessels			percentage	90
Length of newly hatched larvae			mm	4.8–5.0

**Table 2:** Propagation data for common carp in hatchery conditions. Data collected from [27,32,33].

Specification	Unit	Common Carp
Propagation temperature	°C	20–24
Water flow rate (per kg/broodstock)	liters/min	1.0–1.5
Amount of swollen eggs to be incubated in 7 L Zug jar	(a) Number	120–200,000
	(b) Volume (L)	1.5–2.5
Water flow in Zug jar	liters/min	0.5–2.0
Keeping of larvae in	(a) Time	3–4 days
200 L containers	(b) Number	400–600,000
Water flow in container	liters/min	10–15

**Table 3:** Results of advanced fry rearing of common carp in small ponds. Data collected from [27,32,33].

Specification	Common Carp
Duration of rearing	3–4 weeks
Rearing site:	Pond: 100–1000 m^2^
First feed in hatchery	(a) Hard-boiled egg
	suspension
	(b) Newly hatched
	Artemia nauplii
	(c) Collected rotifers
Size of first feed	100–200 µm
Stocking density in rearing pond	100–600 larvae/m^2^
Size of fry at the end of rearing	2.5–3.0 cm
	0.2–0.3 g
Percentage survival:	30–40%

## Data Availability

Data origin from our previous publications cited.
